# Age-Related Decline in Pragmatic Reasoning of Older Adults

**DOI:** 10.1093/geroni/igab046.2619

**Published:** 2021-12-17

**Authors:** W Quin Yow, Jia Wen Lee, Xiaoqian Li

**Affiliations:** Singapore University of Technology & Design, Singapore, Singapore

## Abstract

As speech is often ambiguous, pragmatic reasoning—the process of integrating multiple sources of information including semantics, ostensive cues and contextual information (Bohn & Frank, 2019)—is essential to understanding a speaker’s intentions. Despite current literature suggesting that certain social cognitive processes such as gaze-processing (Slessor et al., 2014) appear to be impaired in late adulthood, it is not well understood if pragmatic reasoning decline with age. Here, we examined young adults’ (aged 19-25; n=41) and older adults’ (aged 60-79; n=41) ability to engage in pragmatic reasoning in a cue integration task. In Experiment 1, participants had to integrate contextual (participants and speaker knew there were two novel objects but the latter could only see one), semantic (“There’s the [novel-label]” or “Where’s the [novel-label]”), and gaze (speaker looked at the mutually-visible object) cues to identify the referent (Nurmsoo & Bloom, 2008). In Experiment 2, participants received contextual and semantic cues less gaze cue. In both experiments, the target referent object for “There” and “Where” trials was the mutually-visible object and the object the speaker could not see respectively. Overall, young adults outperformed older adults, even in the simpler two-cue Experiment 2 (ps<.006). While older adults were significantly above chance in “There” trials for both experiments as well as “Where” trials in Experiment 2 (ps<.05), they had specific difficulty in integrating three cues in “Where” trials, where a more sophisticated interpretation of the multiple cues was required (p=.42). Our findings provide important insights into an age-related decline of pragmatic reasoning in older adults.

